# Cerebrospinal fluid in Alzheimer's: A precious tool

**DOI:** 10.18632/oncotarget.14157

**Published:** 2016-12-24

**Authors:** Lucilla Parnetti, Claudia Cicognola

**Affiliations:** Department of Psychiatry and Neurochemistry, Institute of Neuroscience and Physiology, University of Gothenburg, Sweden

**Keywords:** Alzheimer's disease, cerebrospinal fluid, biomarker, lumbar puncture, diurnal variation, Neuroscience

Alzheimer’s disease (AD) represents the most common neurodegenerative disease leading to dementia, and its prevalence is increasing every year, thus representing a major health and socioeconomic issue. AD pathology affects the brain several years before any of the clinical symptoms appears; therefore, early diagnosis is highly recommended in order to start the treatment as soon as possible, especially in view of the upcoming disease-modifying drugs [1]. To this purpose, diagnostic accuracy is crucial. At present, AD diagnosis is based on combining clinical symptoms, objectified by means of neuropsychological testing, with pathophysiological biomarkers reflecting AD pathology, which can be biochemical (cerebrospinal fluid) or imaging markers (MRI, PET) [2]. In this context, cerebrospinal fluid (CSF) represents a precious tool, since it closely reflects brain pathological processes. This is a great advantage with respect to the blood, where many more confounding compounds are present. Classical CSF biomarkers are represented by peptides and proteins associated with neurodegeneration such as amyloid-beta 1-42 (Aβ42), a misfolding protein that represents the main component of cortical amyloid plaques, and phosphorylated and total tau (p-tau, t-tau), an axonal protein that in AD forms aggregates called neurofibrillary tangles. Aβ42 is present in lower concentrations in AD CSF, due to its accumulation in the cortical plaques, while t-tau and p-tau are increased due to the axonal disruption and release of the proteins in the CSF circulation. Large evidence has been collected about the reliability of these biomarkers in supporting AD diagnosis, thus, they have been introduced in routine AD diagnostics [3]. CSF is collected through lumbar puncture, a medical procedure largely proved to be safe, so that it is routinely and successfully performed in the clinic by trained physicians [4]. A large amount of data has also been collected on the pre-analytical and analytical confounding factors that may affect the measurements of the AD biomarkers in CSF, and guidelines have been produced about its collection, handling and processing [5]. Among the pre-analytical factors, time of day at CSF withdrawal was reported to influence the concentration of Aβ42 in young individuals, as it was reported an increasing trend in Aβ42 over daytime [6]. Subsequent studies, however, disproved this hypothesis, even when other biomarkers such as tau were analysed [7]. Our group carried out a study on neurosurgery patients carrying a CSF drainage, and a wide range of classical and candidate biomarkers (Aβ42, t-tau, p-tau, neurogranin, YKL-40, VILIP-1, Aβ38, Aβ40, sAPPα, sAPPβ, apolipoprotein E) was analysed at different time points during the day and after a night’s sleep. No significant fluctuation in any of the biomarkers was reported, confirming CSF diagnostics as a robust and reliable method, unaffected by external factors [8]. There is a huge body of evidence that suggests CSF as the default biomarker for early diagnosis in cases of AD suspect, but also as a tool for differential diagnosis for other neurodegenerative disorders (Lewy body dementia, vascular dementia, atypical parkinsonism, Creutzfeldt- Jakob disease, etc.). Unfortunately, there is still a stigma regarding lumbar puncture, and clinicians often find resistance in patients when they recommend CSF diagnostics. The population should be informed about the minor risks and high benefits they could get from the procedure, and there should be a close collaboration between the specialist and the general practitioner, in order to select the patients that could mostly benefit from an early diagnosis.
